# Intestinal Animalcules

**Published:** 1858-02

**Authors:** 


### Intestinal Animalcules.

In the Bulletin of the *Academie des Sciences*, (Paris,) of the 30th Nov., 1857, we find the following extract from a brochure by P. H. Halmstein, of Stockholm, (extract presented by M. Rayer,)—" A sailor in consequence of an attack of cholera, had suffered for some time with a derangement of the digestive organs, the more prominent symptoms being those common to an intestinal inflammation. In examining under the microscope the puss from a small ulcer of the rectum mixed with the secretions of this portion of the intestine, the author was able to detect between the globules of puss and of blood quite a number animalcules, which he described under the name of *paramecium coli*. He has since seen the same animalcules in a woman attacked with a chronic inflammation of the large intestine. The patient died, and by examination the author found that the animalcules were much more numerous upon portions of the mucous membrane little altered than upon the portion ulcerated, or with the pus from the ulcers. These animalcules live but a short time after they are removed from the intestine. The secretions in which they are contained should be examined, if we hope to detect them, soon after its removal from the bowels.

---

☞ N. F. Powers, M.D., of Thompson, Ga., an alumnus of the Atlanta Medical College—an occasional contributor to our Journal, and a gentleman of considerable attainments in the profession—has been appointed Professor of Physiology, &c., in the Oglethorpe Medical College, Savannah, Ga.

---

☞ Marshall Hall, Esq., a son of the late Dr. Marshall Hall, is the editor of a work by his father and just published in London, entitled "Prone and Postural Respiration in Drowning and other Forms of Apnœa or Suspended Respiration." The volume contains 216 pages.—*Western Lancet.*

---

☞ The Messrs. Lippincott & Co., of Philadelphia, are about to publish a translation of Malgaigne's great work on Fractures and Dislocations. The translator is Dr. John H. Packard, of that city.—*Ibid.*

